# A-type lamins bind both hetero- and euchromatin, the latter being regulated by lamina-associated polypeptide 2 alpha

**DOI:** 10.1101/gr.196220.115

**Published:** 2016-04

**Authors:** Kevin Gesson, Philipp Rescheneder, Michael P. Skoruppa, Arndt von Haeseler, Thomas Dechat, Roland Foisner

**Affiliations:** 1 Department of Medical Biochemistry, Medical University of Vienna, A-1030 Vienna, Austria;; 2 Center for Integrative Bioinformatics Vienna, Max F. Perutz Laboratories (MFPL), Medical University of Vienna and University of Vienna, Vienna Biocenter (VBC), A-1030 Vienna, Austria;; 3 Bioinformatics and Computational Biology, Faculty of Computer Science, University of Vienna, A-1030 Vienna, Austria

## Abstract

Lamins are components of the peripheral nuclear lamina and interact with heterochromatic genomic regions, termed lamina-associated domains (LADs). In contrast to lamin B1 being primarily present at the nuclear periphery, lamin A/C also localizes throughout the nucleus, where it associates with the chromatin-binding protein lamina-associated polypeptide (LAP) 2 alpha. Here, we show that lamin A/C also interacts with euchromatin, as determined by chromatin immunoprecipitation of euchromatin- and heterochromatin-enriched samples. By way of contrast, lamin B1 was only found associated with heterochromatin. Euchromatic regions occupied by lamin A/C overlap with those bound by LAP2alpha, and lack of LAP2alpha in LAP2alpha-deficient cells shifts binding of lamin A/C toward more heterochromatic regions. These alterations in lamin A/C-chromatin interactions correlate with changes in epigenetic histone marks in euchromatin but do not significantly affect gene expression. Loss of lamin A/C in heterochromatic regions in LAP2alpha-deficient cells, however, correlated with increased gene expression. Our data show a novel role of nucleoplasmic lamin A/C and LAP2alpha in regulating euchromatin.

The confined space of metazoan cell nuclei requires chromatin to be tightly packed while maintaining a high degree of organization to provide genome stability and coordinated operation of the transcriptional machinery (Cremer and Cremer 2010; Bickmore and van Steensel 2013). Conceptually, gene-rich and transcriptionally active chromatin localizes to the nuclear interior, while gene-poor and transcriptionally repressed chromatin preferentially resides at the nuclear periphery (Towbin et al. 2013; Amendola and van Steensel 2014).

The nuclear lamina, a scaffold structure at the periphery of metazoan nuclei, has a major role in chromatin organization by anchoring heterochromatin (Amendola and van Steensel 2014; Gruenbaum and Foisner 2015). Lamins, type V intermediate filaments, are the major constituents of the lamina (Gruenbaum and Foisner 2015). They are categorized into A-type lamins, comprising the major isoforms lamin A and C in mammals, and B-type lamins, including lamin B1 and B2. Whereas B-type lamins are ubiquitously expressed throughout development, A-type lamins are expressed in a differentiation-dependent manner (Dechat et al. 2010a; Gruenbaum and Foisner 2015). Lamins interact with a plethora of proteins in the inner nuclear membrane, which constitute important additional components of the nuclear lamina (Wilson and Berk 2010; Wilson and Foisner 2010; Korfali et al. 2012; de Las Heras et al. 2013).

At the molecular level, genome-wide interactions of chromatin with the nuclear lamina have first been mapped by the DamID technique (Greil et al. 2006) identifying genomic regions attached to the nuclear periphery, so-called lamina-associated domains (LADs). LADs cover nearly 40% of the human genome and are up to 10 Mb long, gene-poor, and enriched in repressive histone marks H3K9me3 and H3K27me3 (Guelen et al. 2008; Peric-Hupkes et al. 2010; Meuleman et al. 2013; van Steensel and Kind 2014; Amendola and van Steensel 2015). These studies led to the concept that the lamina anchors heterochromatin at the nuclear periphery, thereby contributing to gene repression (Towbin et al. 2013; Amendola and van Steensel 2014). Solovei et al. showed that two protein complexes of the lamina redundantly link heterochromatin to the nuclear periphery, the inner nuclear membrane (INM) protein lamin B receptor (LBR), most likely in a complex with B-type lamins, and a complex of A-type lamins with LAP-Emerin-MAN1 (LEM) domain proteins of the INM (Solovei et al. 2013). LBR binds to H3K9me3 via the chromobox 5 (CBX5) protein (Ye and Worman 1996) and to H4K20me2 directly via its Tudor domain (Hirano et al. 2012). LEM proteins contain a bihelical structural motif, the LEM domain that mediates association with chromatin via Barrier-to-Autointegration Factor (BANF1) (Brachner and Foisner 2011). Most LEM proteins are integral components of the INM and interact with lamins (Wilson and Foisner 2010) or require A-type lamins for their proper localization (Vaughan et al. 2001; Brachner et al. 2005). A well-studied group among mammalian LEM proteins comprises isoforms of lamina-associated polypeptide (LAP) 2, encoded by thymopoietin (*Tmpo*, also known as *LAP2*). The major LAP2 isoform in the INM, LAP2beta binds chromatin-associated BANF1 and DNA via its LEM and LEM-like domains, respectively (Cai et al. 2001), and contributes to gene repression by binding to transcriptional repressors germ cell-less (Nili et al. 2001) and ZBTB7B together with histone deacetylase (HDAC) 3 (Somech et al. 2005; Zullo et al. 2012).

Unlike B-type lamins, A-type lamins are also found in a mobile pool (Moir et al. 2000) throughout the nuclear interior (Dechat et al. 2010b). B-type lamins are tightly associated with the INM through post-translational addition of a hydrophobic farnesyl group to their C terminus (Gruenbaum and Foisner 2015). In contrast, lamin C is not farnesylated at all, and pre-lamin A is only transiently farnesylated, as a C-terminal peptide, including the farnesyl group, is removed in a final processing step (Sinensky et al. 1994; Pendas et al. 2002; Barrowman et al. 2008). Thus, A-type lamins can dissociate from the membrane into the nucleoplasm, where they interact with the non-membrane-bound LAP2 isoform, LAP2alpha (Dechat et al. 2000). Deletion of exon 4 of the *Tmpo* gene generated mice specifically lacking LAP2alpha and leads to the selective loss of nucleoplasmic lamin A/C (Naetar et al. 2008). Loss of LAP2alpha causes tissue-specific phenotypes, including increased proliferation of tissue progenitor cells in epidermis, colon, and the hematopoietic system (Naetar et al. 2008), delayed skeletal muscle differentiation (Gotic et al. 2010b), and impaired heart function (Gotic et al. 2010a). However, the molecular mechanisms remain elusive.

In view of the recently reported role of A-type lamins and LEM proteins in anchoring heterochromatin at the nuclear periphery (Solovei et al. 2013), we hypothesized that complexes of A-type lamins and LAP2alpha may also associate with chromatin in the nuclear interior. LAP2alpha has several means to interact with chromatin and DNA, including a LEM and LEM-like motif (Gesson et al. 2014), and was recently reported to associate with chromatin in vivo on a genome-wide level (Zhang et al. 2013).

Using various protocols for chromatin immunoprecipitation (ChIP) in immortalized wild-type (*Tmpo* WT) and LAP2alpha-deficient (*Tmpo* KO) murine dermal fibroblasts (imMDFs), we found that A-type lamins not only associate with heterochromatic LADs as reported (Meuleman et al. 2013; Lund et al. 2014; van Steensel and Kind 2014) but also show a genome-wide interaction with euchromatic regions outside of LADs. Interestingly, euchromatic regions bound by lamin A/C largely overlap with those bound by LAP2alpha and were significantly rearranged and shifted toward heterochromatic regions in *Tmpo* KO cells resulting in changes in gene expression and epigenetic histone marks.

## Results

### A-type lamins interact with both heterochromatic LAD and euchromatic inter-LAD regions

**Figure 1.**
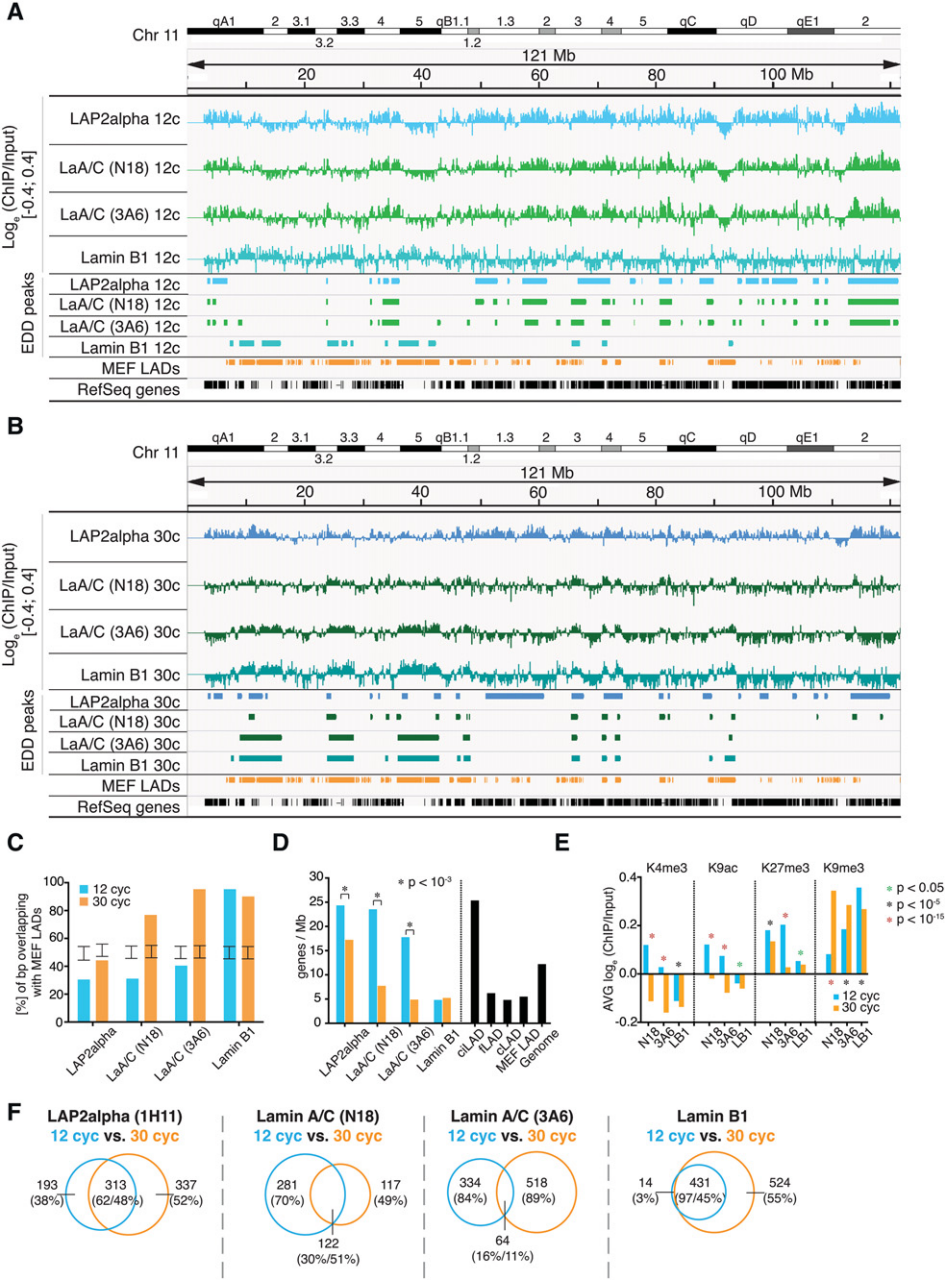
LAP2alpha and Lamin A/C associate with eu- and heterochromatin. (*A*,*B*) Screenshot of Integrative Genomics Viewer (IGV) tracks of Chromosome 11 (mm9) representing log_e_ ratios of ChIP/Input, scale is [−0.4;0.4] (*upper* panel); peak regions identified by EDD (*mid* panel); MEF LADs (GSE36132) and the RefSeq gene track (*lower* panel). Chromatin was sonicated for (*A*) 12 or (*B*) 30 cycles. ChIPs were performed with antibodies against LAP2alpha (8C10-1H11) and lamin A/C (3A6-4C11 and N18). (*C*) MEF LAD overlap. Degree of overlap (% of bp) of EDD peaks with MEF LADs. Error bars indicate the interval that contains 95% of all mean overlaps obtained through random permutation tests. All overlaps are significantly smaller or larger than expected under the null model (*P* < 10^−4^). (*D*) Gene density. Average gene density in EDD/DamID peak regions. Significance of gene density change from 12- to 30-cycle samples was tested using the Wilcoxon rank-sum test. (*) *P* < 10^−3^. (*E*) Histone marks in lamin-interacting sites. Average log_e_ (ChIP/Input) of H3K4me3, H3K9ac, H3K27me3, and H3K9me3 in regions associated with lamin A/C and lamin B1. Differences between distributions of values from 12- to 30-cycle samples were tested by the Wilcoxon rank-sum test; green asterisk signifies *P* < 0.05, black asterisk *P* < 10^−5^, and red asterisk *P* < 10^−15^. (*F*) Extent of overlap between EDD peaks of LAP2alpha, lamin A/C, and lamin B1 after 12 and 30 cycles of sonication; numbers indicate Mb.

Lamins associate with heterochromatic LADs at the nuclear periphery (Guelen et al. 2008; Peric-Hupkes et al. 2010; Kind et al. 2013; van Steensel and Kind 2014). To test whether nucleoplasmic lamin A/C and LAP2alpha also interact with chromatin, we performed ChIP for LAP2alpha and for two distinct domains of lamin A/C, using antibodies N18, directed against lamin A/C's N terminus, and 3A6, detecting the C-terminal Ig fold. As a control for peripheral lamina-chromatin interactions, we performed lamin B1 ChIP. Our ChIP protocol applied moderate sonication (12 cycles in a Bioruptor) yielding DNA fragments of 100–500 bp and allowing efficient immunoprecipitation of lamins and LAP2alpha (Supplemental Fig. 1). ChIP and respective input samples were analyzed by deep sequencing (ChIP-seq). Integrative Genomics Viewer (IGV) tracks, representing the log_e_ (ChIP/input) signal, display wide-range chromatin interactions of all proteins (Fig. 1A). The enriched domain detector (EDD) peak-calling algorithm (Lund et al. 2014) identified around 250 EDD peaks in lamin A/C, LAP2alpha, and lamin B1 ChIP samples with an average length of 1–2 Mb, in total covering 15%–20% of the genome (Table 1). The SICER algorithm (Xu et al. 2014) identified significantly more (7000–9000) and smaller (average size of 25 kb) regions to be associated with LAP2alpha, lamin A/C, and lamin B1 (Table 1). Regions with a high SICER peak density overlapped with EDD peaks (Supplemental Fig. 2).

**Table 1.**
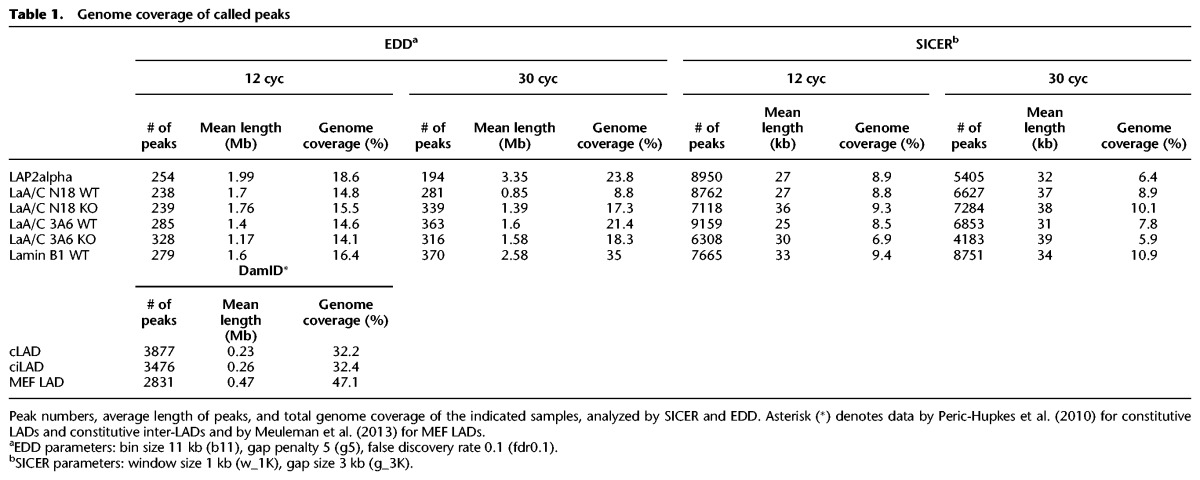
Genome coverage of called peaks

Next, we tested whether the lamin A/C-, LAP2alpha-, and lamin B1-enriched chromatin domains match published lamin B1 LADs in mouse embryonic fibroblasts (MEF) identified by DamID (Meuleman et al. 2013). Whereas 95% of the lamin B1-associated genomic regions overlapped with lamin B1-LADs, only 27%–41% of lamin A/C- and LAP2alpha-associated regions overlapped (Fig. 1A,C). Accordingly, in contrast to LADs, lamin A/C- and LAP2alpha- EDD peaks were gene-rich (18–24 genes/Mb versus 5 genes/Mb in LADs) (Fig. 1A,D) and enriched in active H3K4me3 and H3K9ac histone marks, as well as in H3K27me3 (Fig. 1E). Lamin B1 predominantly associated with gene-poor regions (Fig. 1D), devoid of active histone marks and maintaining the highest levels of the heterochromatic H3K9me3 mark (Fig. 1E). Thus, whereas lamin B1 interacted mainly with heterochromatic LADs, lamin A/C and LAP2alpha surprisingly occupied active, euchromatic regions of the genome.

Elucidating the disparity between our data and the reported lamin A/C binding to LADs (Meuleman et al. 2013; van Steensel and Kind 2014), we noted that lamin A/C ChIP samples obtained after moderate sonication (12 cycles) contained, in addition to the main pool of DNA fragments of ∼350 bp, a significant fraction of larger fragments (∼1.5 kb) (Supplemental Fig. 1). This fraction likely represents heterochromatic genomic regions, which were excluded from Illumina sequencing due to size selection. In order to make these regions accessible to analysis, we extended the sonication to 30 cycles and reperfomed the ChIP. This resulted in a clear reduction of the larger DNA fragment pool, while still allowing for efficient immunoprecipitation (Supplemental Fig. 1). Under these conditions, lamin A/C-3A6-associated chromatin regions are shifted toward gene-poor heterochromatin and overlap with LADs by ∼96%, resembling lamin B1-associated regions (Fig. 1B–D). Concomitantly, active histone marks are lost and heterochromatic H3K9me3 is increased in lamin A-3A6-recovered regions (Fig. 1E). Moreover, lamin A/C-3A6 EDD peaks of the 30-cycle sample show only minimal overlap (11%) with peaks obtained from the 12-cycle sample, while the lamin B1 peaks overlapped nearly completely, although the recovery of lamin B1 LADs was significantly improved in the 30- versus 12-cycle preparation (Fig. 1F). Similar effects were observed when comparing lamin A/C-N18-associated chromatin regions in 30- versus 12-cycle ChIP preparations (Fig. 1C–F). Taken together, these results suggest that A-type lamins occupy both heterochromatic LADs and euchromatic regions outside of LADs, while lamin B1 is enriched in heterochromatic LADs only.

### LAP2alpha and lamin A/C occupy similar genomic regions in euchromatin

**Figure 2.**
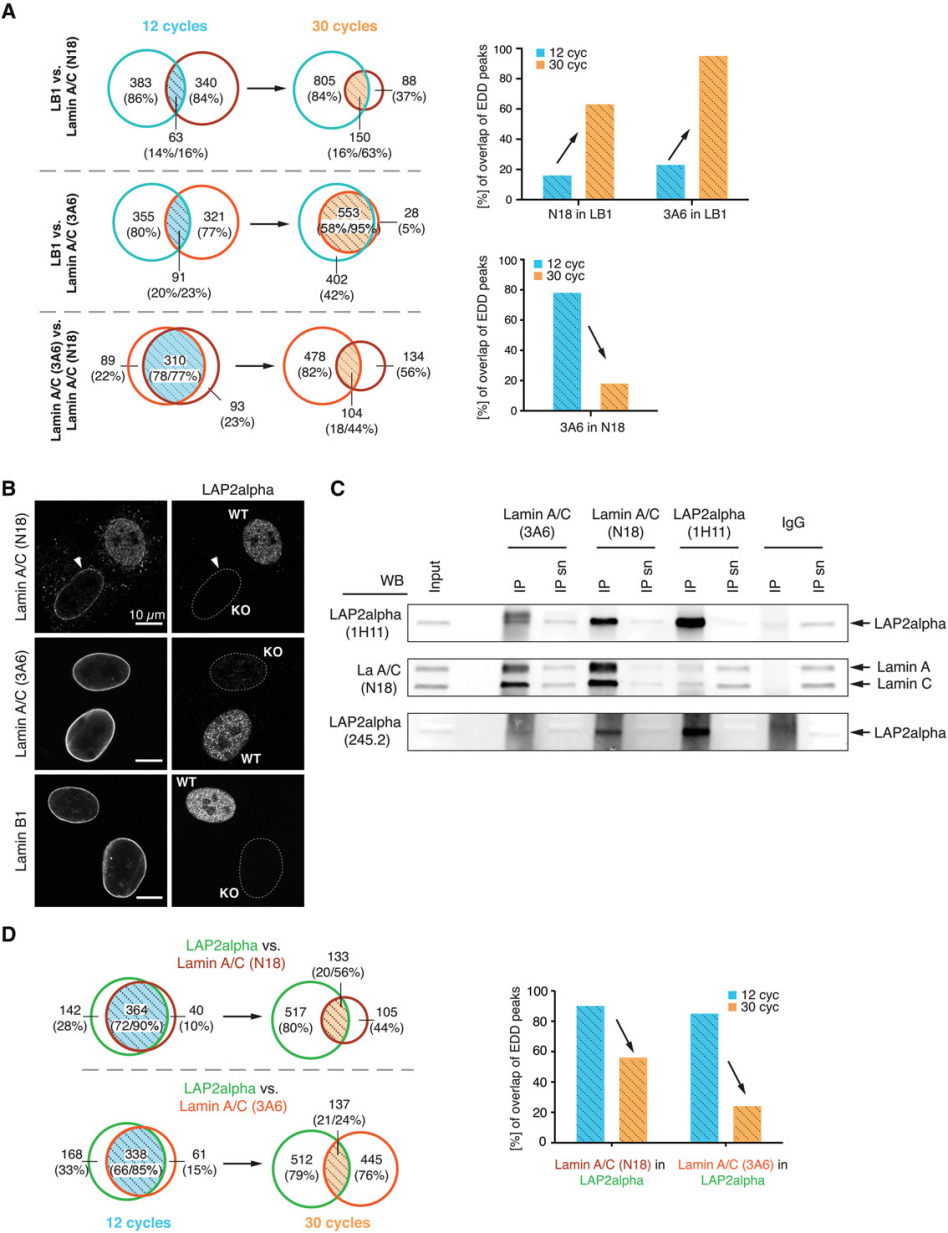
LAP2alpha and Lamin A/C overlap in euchromatic regions. (*A*) Venn diagrams of genome-wide overlapping EDD peaks (Mb) of lamin B1 and lamin A/C samples upon 12 and 30 cycles of sonication. Overlaps (hatched) of lamin A/C-3A6 and -N18 with lamin B1 and overlaps of lamin A/C-3A6 with -N18 are highlighted in light blue (12 cycles) and light orange (30 cycles), and the corresponding % of overlap of EDD peaks are depicted as bar graphs. (*B*) Confocal immunofluorescence microscopic images of a mixed culture of *Tmpo* WT and KO mouse dermal fibroblasts double-stained for LAP2alpha and lamins as indicated. Unstained nuclei in LAP2alpha panels are outlined by a white dashed line. Scale bar, 10 µm. (*C*) Co-IPs for lamin A/C-3A6, lamin A/C-N18, and LAP2alpha were performed in *Tmpo* WT imMDFs. Normal rabbit IgG was used as a negative control. (*D*) Venn diagrams of genome-wide overlapping EDD peaks (Mb) of LAP2alpha and Lamin A/C samples upon 12 and 30 cycles of sonication. Overlaps (hatched) of LAP2alpha with lamin A/C-3A6 and -N18 are highlighted in light blue (12 cycles) and light orange (30 cycles), and the corresponding % of overlap of EDD peaks are depicted as bar graphs.

Our data showed that sonication conditions are a critical parameter for the nature of recovered lamin A/C-associated chromatin regions: moderate sonication (12 cycles) enriching for euchromatic regions, and extended sonication (30 cycles) for heterochromatic LADs. Intriguingly, this effect was more pronounced in the lamin A/C-3A6 compared to lamin A/C-N18 ChIP samples. Whereas in the 12-cycle ChIP, the lamin A/C-associated chromatin regions retrieved by the two lamin A/C antibodies coincided by nearly 80%, they considerably differed in the 30-cycle ChIP (Fig. 2A, lower panel), indicating that the 3A6 and N18 antibodies target different pools of A-type lamins. However, the overall overlap of lamin A/C-associated genomic regions with lamin B1-associated LADs strongly increased in the 30- versus 12-cycle preparation for both antibodies, N18 and 3A6 (Fig. 2A, bar graph).

The two lamin A/C ChIP samples also differ in their epigenetic marks. Lamin A/C-N18-associated domains contained more active histone marks (H3K4me3, H3K9ac) and less repressive H3K9me3 than the lamin A/C-3A6-bound regions in euchromatin-enriched samples, and they had more H3K27me3 marks in the heterochromatin-enriched samples (Fig. 1E). These data suggest that antibody 3A6 targets lamin A/C associated with heterochromatin, as opposed to antibody N18 that may preferentially detect lamin A/C complexes in an open chromatin context. This hypothesis was further supported by immunofluorescence analyses of mixed cultures of *Tmpo* WT and *Tmpo* KO fibroblasts. While lamin A/C-N18 detects both peripheral and nucleoplasmic lamin A/C pools in *Tmpo* WT (but not in *Tmpo* KO) cells, lamin A/C-3A6 and lamin B1 detect primarily the peripheral lamina in both cell types (Fig. 2B). Furthermore, coimmunoprecipitation of lamin A/C and LAP2alpha revealed that lamin A/C-N18-antibody coprecipitated considerably more LAP2alpha than lamin A/C-3A6 antibody (Fig. 2C). We concluded that antibody 3A6 mainly detects lamin A/C complexes not bound to LAP2alpha, whereas antibody N18 targets nucleoplasmic lamin A/C in complexes with LAP2alpha.

Having shown that lamin A/C binds in a heterochromatic and a euchromatic environment, we next tested to what extent the lamin A/C binding sites correlate with those of LAP2alpha. By overlapping of lamin A/C- and LAP2alpha-associated regions, we found that, in euchromatin-enriched samples (12 cycles), the majority of lamin A/C EDD peaks (85% for 3A6 and 90% for N18) concurred with LAP2alpha EDD peaks (Fig. 2D). After extended sonication (30 cycles), the congruency of LAP2alpha with lamin A/C-3A6 and -N18 is diminished to 21% and 20%, respectively. Thus, the overlap of lamin A/C- and LAP2alpha-associated chromatin regions decreased markedly in 30- versus 12-cycle preparations (Fig. 2D, bar graph). However, still more than half (56%) of lamin A/C-N18-associated chromatin (but only 24% of 3A6-associated regions) is also bound by LAP2alpha under these conditions (Fig. 2D). Similar results were obtained by comparing the number of SICER peaks per Mb bin between LAP2alpha versus lamin A/C-3A6 and LAP2alpha versus lamin A/C-N18 ChIP samples (Supplemental Fig. 2B). Furthermore, sequential ChIP of lamin A/C and LAP2alpha followed by qPCR revealed that lamin A/C and LAP2alpha co-occupy various genomic regions only in the euchromatin-enriched sample (Supplemental Fig. 3). Overall, we concluded that LAP2alpha and lamin A/C occupy similar genomic regions under conditions enriching for euchromatin, whereas in heterochromatin-enriched regions, lamin A/C-chromatin association seems to be mostly independent of LAP2alpha.

### Lamin A/C-binding is reorganized toward heterochromatic regions in LAP2alpha-deficient cells

**Figure 3.**
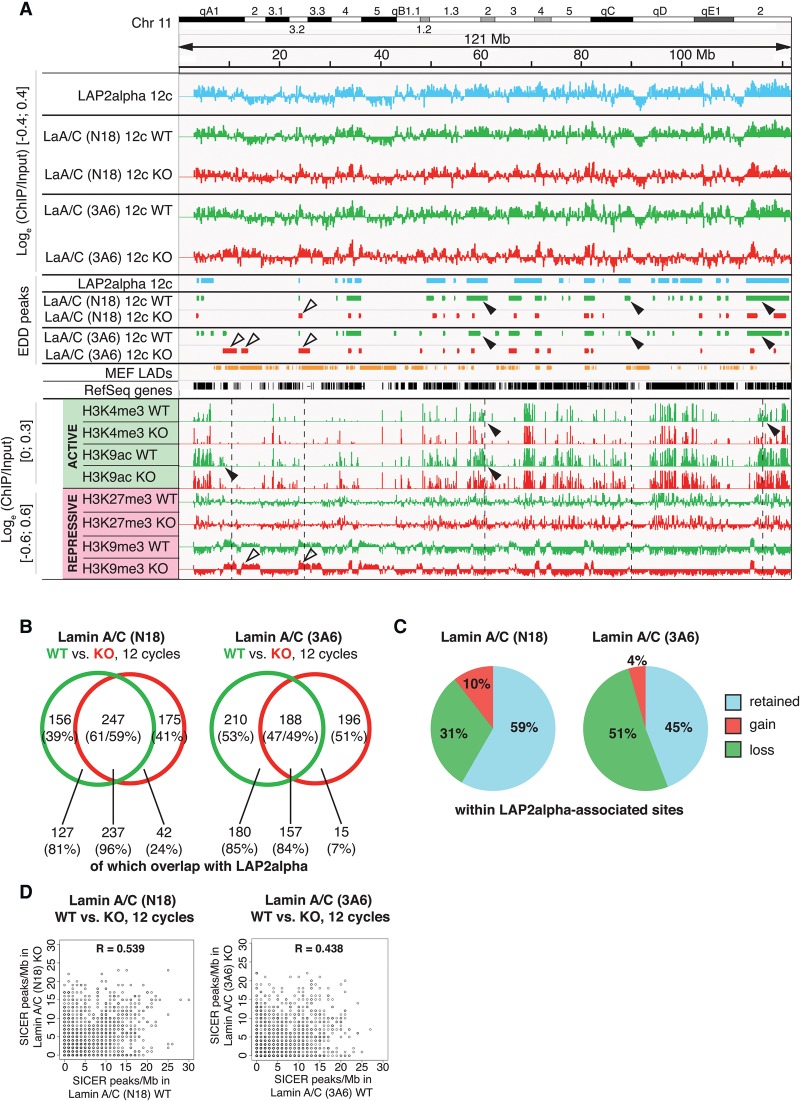
Lamin A/C-chromatin associations are rearranged in LAP2alpha-deficient versus wild-type cells. (*A*) Screenshots of IGV tracks of Chromosome 11 (mm9) representing log_e_ (ChIP/Input) [−0.4;0.4], peak regions identified by EDD, MEF LADs, the RefSeq gene track, and histone mark tracks. Shown are data for LAP2alpha and lamin A/C (precipitated with 3A6 or N18 antibodies in *Tmpo* WT and KO cells) after 12 cycles of sonication and data of histone marks after 12 cycles of sonication (scale for active marks H3K4me3 and H3K9ac is log_e_ (ChIP/Input) [0;0.3] and for repressive marks H3K27me3 and H3K9me3 log_e_ (ChIP/Input) [−0.6;0.6]). Black arrowheads point to regions of loss of lamin A/C or histone marks and white arrowheads to regions of gain of lamin A/C or histone marks in *Tmpo* KO cells. (*B*) Venn diagrams of EDD peak overlaps [Mb] between lamin A/C in *Tmpo* WT and KO samples after 12 cycles of sonication. Numbers *below* Venn diagram show overlap (in Mb and %) of lamin A/C EDD peak fractions (occurring only in WT, in WT and KO, or KO only) with LAP2alpha EDD peaks. (*C*) Redistribution of lamin A/C within LAP2alpha-associated sites. Pie chart depicting percentage of lamin A/C-associated regions (identified by antibodies N18 or 3A6) within the LAP2alpha-associated regions that retained, lost, or gained lamin A/C binding in *Tmpo* KO versus WT samples. (*D*) SICER peak correlations (peaks/Mb) corresponding to data in *B*.

Given that LAP2alpha and lamin A/C predominantly co-occur in euchromatic genomic regions, we next aimed to investigate the role of LAP2alpha in lamin A/C chromatin interaction by extending our ChIP-seq experiments to *Tmpo* KO fibroblasts (Supplemental Fig. 4). We assessed changes in lamin A/C EDD peaks between *Tmpo* WT and *Tmpo* KO samples on a genome-wide scale in euchromatin-enriched regions. This analysis revealed lamin A/C-associated chromatin regions exclusively in *Tmpo* WT cells (“lost in KO”) (Fig. 3A, black arrowheads) or exclusively in *Tmpo* KO cells (“gained in KO”) (Fig. 3A, white arrowheads). At the genome-wide level, only 61% of the lamin A/C-N18- and 47% of the lamin A/C-3A6-bound regions were detected in both *Tmpo* WT and KO cells (“WT^KO”); the remaining regions were either lost (39% of N18 and 53% of 3A6 peaks) or gained (41% of N18 and 51% of 3A6 peaks) in *Tmpo* KO cells (Fig. 3B). Interestingly, regions that lost or retained lamin A/C interaction in the *Tmpo* KO cells overlap with LAP2alpha-associated regions by 81%–96%, while the newly gained lamin A/C associated regions showed only 24% (N18) and 7% (3A6) overlap (Fig. 3B). This indicates a reorganization and partial loss of lamin A/C within the LAP2alpha-associated regions in *Tmpo* WT versus KO cells and a gain of lamin A/C binding primarily outside these regions. Within the LAP2alpha-associated regions, 45%–59% of lamin A/C-associated regions were retained in the *Tmpo* KO cells, while 31%–51% were lost and only a marginal fraction was gained (Fig. 3C). This rearrangement of lamin A/C-chromatin association is also reflected by the low correlation of SICER peak densities in *Tmpo* WT versus KO cells (R = 0.539 for N18 and R = 0.438 for 3A6) (Fig. 3D).

Interestingly, partial reorganization of lamin A/C-N18- and lamin A/C-3A6-associated domains in *Tmpo* KO versus WT cells was also detectable in the 30-cycle ChIP analyses (Supplemental Fig. 5), suggesting that, in the absence of LAP2alpha, the association of lamin A/C with chromatin is reorganized at a genome-wide level.

**Figure 4.**
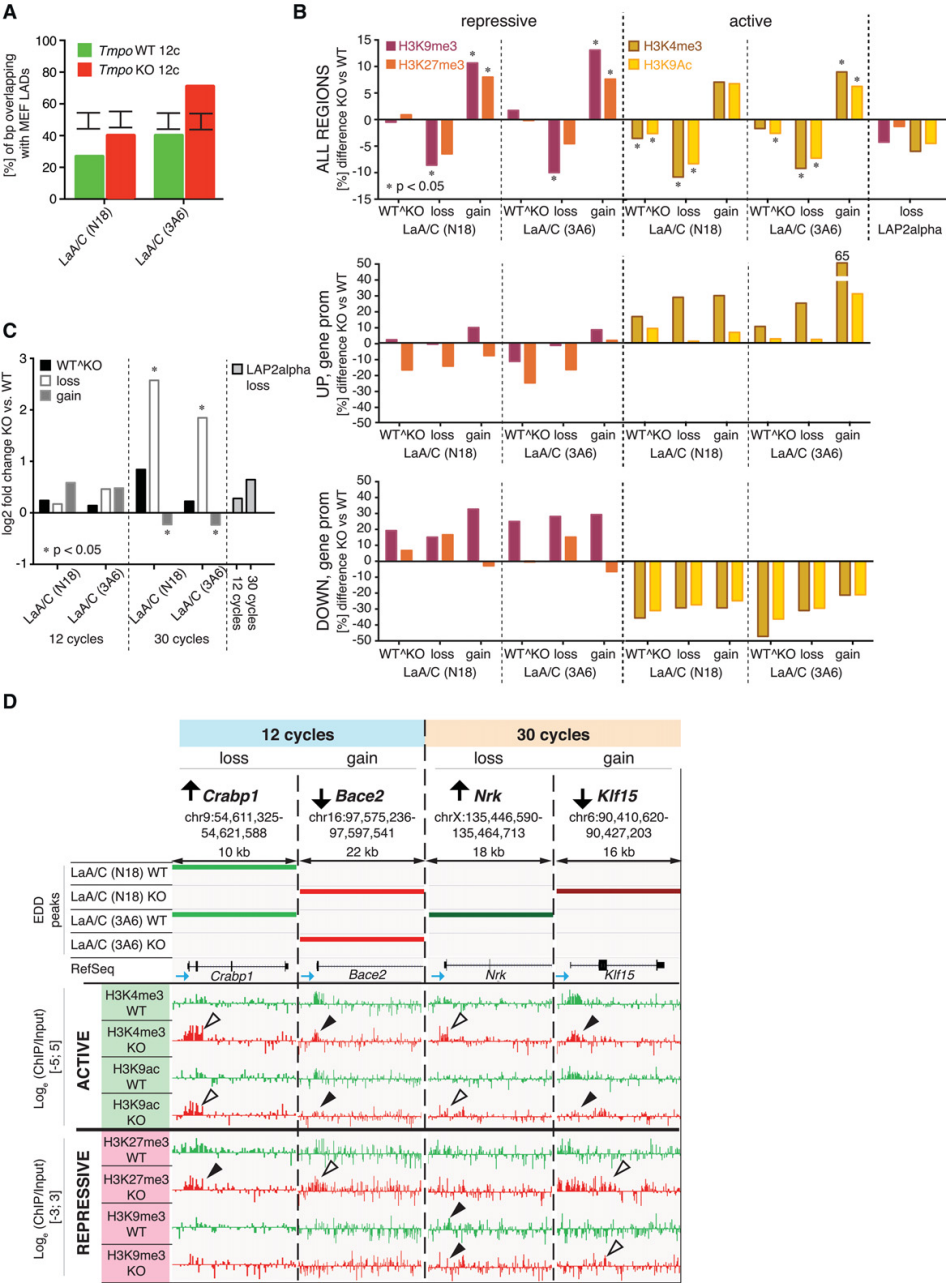
Consequences of lamin A/C redistribution in LAP2alpha-deficient cells on chromatin organization and gene expression*.* (*A*) MEF LAD overlap. Degree of overlap (% of bp) of EDD peaks obtained after 12 cycles of sonication for lamin A/C-N18 and -3A6 in *Tmpo* WT and KO mouse fibroblasts with MEF LADs. Error bars indicate the interval that contains 95% of all mean overlaps obtained through random permutation tests. All overlaps are significantly smaller or larger than expected under the null model (*P* < 10^−4^). (*B*) Change in repressive and active histone marks. EDD peak regions of lamin A/C (N18 and 3A6) in *Tmpo* WT and KO cells were divided into regions occurring in both samples (“WT^KO”), losing association in *Tmpo* KO (“loss”), and gaining associations *Tmpo* KO (“gain”). Percent difference (*Tmpo* KO vs. WT) in the abundance of repressive (H3K27me3, H3K9me3) and active (H3K4me3, H3K9ac) histone marks present in all lamin A/C-N18 and -3A6 “WT^KO,” “loss,” and “gain” regions, all regions of LAP2alpha loss (ALL REGIONS) and promoter regions of up- (UP, gene promoter) and down-regulated (DOWN, gene promoter) in lamin A/C-N18 and -3A6 “WT^KO,” “loss,” and “gain” regions, after 12 sonication cycles. Asterisk (*) denotes significant change in histone mark abundance (*P* < 0.05) compared to random permutation testing. (*C*) Gene expression change. Average log_2_-fold change of differentially regulated genes in lamin A/C-N18 and -3A6 “WT^KO,” “loss,” and “gain” regions and regions of LAP2alpha loss after 12 and 30 sonication cycles. Asterisk (*) denotes significant gene expression change (*P* < 0.05) compared to random permutation testing. (*D*) Redistribution of lamin A/C upon loss of LAP2alpha alters gene expression and histone marks on promoters of affected genes. IGV track compilation of up- and down-regulated genes in regions of loss and gain of lamin A/C in euchromatin-enriched (12 cycle) and heterochromatin-enriched (30 cycle) regions. Shown are EDD peak tracks for lamin A/C-N18 and -3A6 in *Tmpo* WT and KO cells obtained after 12 and 30 cycles of sonication, followed by RefSeq tracks of the respective genes (blue arrow denotes direction of transcription) and histone mark data tracks obtained after 12 cycles of sonication; scale for active marks H3K4me3 and H3K9ac is log_e_ (ChIP/Input) [−5;5] and for repressive marks H3K27me3 and H3K9me3 log_e_ (ChIP/Input) [−3;3]. Black and white arrowheads point to regions of loss and gain of histone marks in *Tmpo* KO, respectively.

In view of our findings that lamin A/C and LAP2alpha co-occupy particularly euchromatic genomic regions, while lamin A/C association with heterochromatic LADs occurs largely independent of LAP2alpha, we hypothesized that loss of LAP2alpha might affect the equilibrium between euchromatic and hetereochromatic lamin A/C pools. Indeed, we found that the overlap of lamin A/C-3A6 EDD peaks with LADs increased from 41% in wild-type to 74% in *Tmpo* KO cells and that of lamin A/C-N18-identified EDD peaks from 27% to 41% (Fig. 4A), indicating that lamin A/C tends to relocalize from euchromatic to heterochromatic regions in the absence of LAP2alpha.

### Rearrangement of lamin A/C-chromatin association in LAP2alpha-deficient cells affects epigenetic profiles and gene expression

To investigate whether the loss of LAP2alpha-chromatin interaction and the rearrangement of lamin A/C on chromatin affects epigenetic marks and gene expression, we analyzed active and repressive histone mark abundance (see histone mark tracks in Fig. 3A) in genomic regions that gained or lost lamin A/C binding in *Tmpo* KO versus WT cells.

Repressive H3K9me3 and H3K27me3 marks accumulated in regions that gained lamin A/C binding (“gain”) and diminished in regions that lost lamin A/C binding (“loss”) in *Tmpo* KO, whereas the abundance of these marks did not change in regions of unaltered lamin A/C-association (“WT^KO”) (Fig. 4B). Intriguingly, also the level of active H3K4me3 and H3K9ac marks decreased in regions that lost lamin A/C-binding in *Tmpo* KO cells and increased in regions that gained lamin A/C binding. Thus, changes in lamin A/C binding to chromatin in *Tmpo* KO versus WT cells affect repressive and active marks similarly. Loss of LAP2alpha binding had a similar effect on the epigenetic profile as the loss of lamin A/C binding (Fig. 4B, upper panel). Changes in epigenetic marks were most prominent in ChIP samples enriched in euchromatin (12 cycles); in heterochromatin-enriched ChIP samples (30 cycles) these marks changed only marginally (Supplemental Fig. 6).

Having determined that loss or gain of lamin A/C binding affects both active and repressive histone marks in a similar manner, we wondered whether these changes are reflected by changes in gene expression. Assessing global gene expression changes in *Tmpo* KO versus WT in imMDFs by RNA-seq, we found 616 genes to be differentially expressed (false discovery rate [FDR] < 0.05), of which 341 were up-regulated and 275 were down-regulated (Supplemental Table 1). Of the 616 de-regulated genes, 239 were located in genomic regions of altered lamin A/C association (Supplemental Table 2). As expected, promoters of up-regulated genes showed an increase in active and a decrease in repressive histone marks and vice versa for down-regulated genes (Fig. 4B, lower panels, 4D; Supplemental Fig. 6). However, in the euchromatin-enriched samples, gain and loss of lamin A/C-gene association in *Tmpo* KO cells did not strictly correlate with overall down- and up-regulation of gene expression, respectively (Fig. 4B,C), although expression of several genes is clearly increased upon loss of lamin A/C- and decreased upon gain of lamin A/C-binding in *Tmpo* KO cells (Fig. 4D). In the heterochromatin-enriched samples (30 cycles), loss of lamin A/C binding was linked to a general increase in gene expression, and gain of lamin A/C binding slightly decreased gene expression (Fig. 4C,D; Supplemental Fig. 6). Loss of LAP2alpha-association correlated with neither up- nor down-regulation of gene expression in both samples.

## Discussion

Most studies performed to date report predominant binding of lamin A/C to heterochromatic regions (Kubben et al. 2012; Lund et al. 2013, 2014; McCord et al. 2013; Meuleman et al. 2013; van Steensel and Kind 2014). We show here that lamin A/C, but not lamin B1, binds to both hetero- and euchromatin.

We found this discrepancy to arise from the extent of chromatin shearing, where moderate conditions preserve and enrich for open euchromatin, while heterochromatin yields larger fragments usually excluded from deep sequencing. Conversely, prolonged sonication enriches heterochromatin, while euchromatin may be lost due to hyperfragmentation. Our findings are in line with recent data showing that sonication versus micrococcal nuclease digestion in lamin A/C ChIP identified only partially overlapping lamin A/C-bound genomic regions (Lund et al. 2015).

Furthermore, the location and accessibility of the lamin A/C epitope recognized by the antibodies used for ChIP can ostensibly affect ChIP results. A lamin A/C antibody against the N terminus (N18) favored lamin A/C complexes containing LAP2alpha, while an antibody to lamin A/C's C-terminal Ig fold (3A6) preferentially precipitated heterochromatin-associated lamin A/C not bound by LAP2alpha. As LAP2alpha interacts with the Ig fold of lamin A/C (Dechat et al. 2000), it may compete with binding of the C-terminal antibody 3A6.

Mature lamin A/C in the nuclear interior colocalizes with LAP2alpha (Dechat et al. 2000; Naetar et al. 2008). As euchromatic regions associated with lamin A/C were largely congruent with those bound by LAP2alpha, it is conceivable that euchromatin-associated lamin A/C is predominantly localized in the nuclear interior, whereas heterochromatin-bound lamin A/C is part of the peripheral lamina. Overall, our data are consistent with a model in which lamin A/C, together with lamin B1, provide a stable anchor for heterochromatin at the nuclear lamina (Solovei et al. 2013; Towbin et al. 2013; Amendola and van Steensel 2014; Gruenbaum and Foisner 2015). Lamin A/C binding to euchromatin in the nuclear interior may be more dynamic, providing a flexible chromatin environment allowing efficient response to epigenetic and transcriptional regulators in a context-dependent manner.

In a LAP2alpha-deficient background, lamin A/C relocalizes toward heterochromatic regions. How can LAP2alpha affect lamin A/C-chromatin interactions? Lamin A/C and LAP2alpha complexes in the nuclear interior are highly mobile (Moir et al. 2000; Dechat et al. 2004) and may thus bind chromatin in a dynamic manner, while immobile lamin A/C structures in the absence of LAP2alpha may bind chromatin more stably. Alternatively, LAP2alpha may compete with lamin A/C for binding to the same chromatin regions, ensuring flexibility of chromatin organization.

What are the consequences of altered lamin A/C chromatin interactions in *Tmpo* KO versus WT cells? Interestingly, in euchromatin both repressive and active histone marks were decreased in regions that lost lamin A/C binding and increased in regions that gained lamin A/C, without affecting overall gene expression. Thus, lamin A/C-LAP2alpha complexes may primarily be involved in establishing a chromatin environment permissive for epigenetic regulation, rather than regulating gene expression directly by binding to promoters as suggested for lamin A/C (Lund et al. 2013). In contrast, in heterochromatin epigenetic marks do not change in regions that gain or lose lamin A/C binding in *Tmpo* KO versus WT cells, but loss of lamin A/C binding correlated with increased gene expression, indicating that lamin A/C may serve different functions in euchromatin versus heterochromatin. The open and transcriptionally active environment in euchromatin allows efficient access of chromatin regulators such as epigenetic modifiers, nucleosomal remodeler complexes, and transcriptional regulators. Lamin A/C-LAP2alpha complexes may contribute to this open chromatin state and thereby favor dynamic chromatin regulation. In such a scenario, gene expression changes occur in a context-dependent manner, for instance, during differentiation due to the presence or absence of specific transcription factors. In contrast, heterochromatin is tightly packed and less accessible for regulators. Lamin A/C in the lamina is known to contribute to heterochromatin formation and stable gene repression (Peric-Hupkes et al. 2010; Kind et al. 2013; Solovei et al. 2013; Harr et al. 2015). Therefore, loss of lamin A/C binding in heterochromatic regions may have profound effects on gene expression.

It is also tempting to speculate that an impairment of lamin's chromatin-regulating functions in the diseases linked to *LMNA* mutations (laminopathies) may contribute to the disease phenotype. In line with this hypothesis, expression of a muscle disease-causing lamin mutant in *Caenorhabditis elegans* impairs cell-type–specific reorganization of repetitive DNA arrays during muscle differentiation (Mattout et al. 2011). In addition, expression of progerin, a lamin A mutant causing the premature aging disease, Hutchinson Gilford Progeria Syndrome (HGPS), affects heterochromatin organization (Shumaker et al. 2006; McCord et al. 2013). Interestingly, while mature lamin A is not farnesylated, progerin retains its farnesyl moiety and is tightly bound to the INM at the nuclear periphery similar to lamin B1 (Dechat et al. 2007). We recently showed that expression of progerin causes loss of nucleoplasmic lamin A/C and LAP2alpha, leading to down-regulation of the expression of extracellular matrix components (Vidak et al. 2015).

## Methods

### Cell culture

Immortalized murine dermal *Tmpo* WT and KO fibroblasts (Naetar et al. 2008) were cultured at 37°C, 5% CO_2_ in Dulbecco's modified Eagle's medium (DMEM), supplemented with 10% FCS, 2 mM L-glutamine, 100 U/mL penicillin, 100 µg/mL Streptomycin, 0.1 mM nonessential amino acids.

### Chromatin immunoprecipitation followed by deep sequencing (ChIP-seq)

ChIP was performed as previously described (Hauser et al. 2002) (see also Supplemental Material). Briefly, cells were cross-linked for 10 min with 1% formaldehyde and chromatin prepared by lysis (2× 10 min on ice). Nuclei-containing pellets were resuspended in lysis buffer (50 mM Tris-HCl [pH 8.1], 10 mM EDTA, 1% sodium dodecyl sulfate [SDS], 1 mM PMSF, 1× complete protease inhibitor) and sonicated with a Bioruptor Plus (Diagenode) for 12 or 30 cycles. For histone mark ChIPs, 12 cycles of sonication were applied. Fifty micrograms of chromatin were used for ChIP. Input chromatin was diluted 1:10 in ChIP dilution buffer (16.7 mM Tris-HCl [pH 8.1], 167 mM NaCl, 1.2 mM EDTA, 1.1 % Triton X-100, 0.01% SDS) and incubated with antibodies overnight. Chromatin-antibody complexes were captured with magnetic protein A/G beads and crosslinks were reversed. Eluted DNA was purified with the ChIP DNA Clean & Concentrator kit (Zymo Research).

Sequencing libraries were prepared from DNA fragments ranging from 100 to 800 bp by the Vienna Biocenter CSF NGS unit (www.csf.ac.at) and sequenced either on an Illumina HiSeq 2000 or HiSeq 2500 at the CSF NGS unit. After de-multiplexing, adapters were clipped with cutadapt (Martin 2011). Reads were mapped to the mouse genome (NCBI37/mm9 annotation from July 2007) with Bowtie 2 version 2.1.0 (Langmead and Salzberg 2012) using default parameters. Reads with a mapping quality below 20 were discarded. As suggested by Lund et al. (2014), reads stemming from PCR duplicates were removed and all ChIP read files were down-sampled to match the read count of their respective input sample using Picard (http://broadinstitute.github.io/picard/). Supplemental Table 3 lists the number of sequenced reads and mapped reads after filtering and de-duplication for all samples.

### ChIP-seq data processing

Regions of enrichment (peaks) were identified using EDD version 1.0.2 (Lund et al. 2014) and SICER version 1.1 (Zang et al. 2009). Since EDD's auto parameter estimation was unsuited for our data sets, we used the parameter set “-b 11 –g 5 –fdr 0.1” (bin size 11 kb, gap penalty 5) suggested for lamin A by Lund et al. (2014). Based on visual inspection, these settings yielded the best results for our data. For SICER, we used a window size of 1000 bp, a gap size of 3000 bp, and a false discovery rate of 0.01, to account for the characteristics of lamin and LAP2alpha binding. See Supplemental Material for more details and Table 1 for information on peak number and genome coverage for all ChIP samples.

### RNA sequencing and analysis

Ten micrograms of purified, rRNA-depleted RNA (>200 bp) were fragmented by hydrolysis (40 mM TrisOAc at pH 8.2, 100 mM KOAc, 150 mM MgOAc) at 94°C for 3 min. First-and second-strand cDNA synthesis was performed as described in detail in the Supplemental Material. Sequencing libraries were prepared by the Vienna Biocenter CSF NGS unit using the NEBNext Library Prep Reagent Set for Illumina (NEB), multiplexed (2 samples/lane), and sequenced on HiSeq 2000 (Illumina) at the CSF NGS unit. RNA-seq data analysis was done as described by Anders et al. (2013) and in the Supplemental Material. All significantly up- or down-regulated genes are listed in Supplemental Table 1. Significant changes in expression in Lamin A/C gain, loss, and WT^KO regions were detected by comparing the actual log_2_-fold change to fold changes obtained from 10,000 random permutations of differentially expressed genes (*P* < 0.05).

### Data viewing

ChIP-seq data (peaks and log ratios) were visualized with the Integrative Genomics Viewer (Robinson et al. 2011). Log ratios (base e) between Input and ChIP samples were computed for nonoverlapping 10-kb bins using EDD-tools (https://github.com/CollasLab/edd). Genes shown in the browser view were obtained from the RefSeq data track provided by the UCSC Genome Browser. cLAD, ciLAD, and MEF LAD annotations were downloaded from the GEO database (accession number GSE17051 [Peric-Hupkes et al. 2010] and GSE36132 [Meuleman et al. 2013]).

### Cross-linked immunoprecipitation

Fifty to one hundred micrograms of cross-linked chromatin, diluted 1:10 in ChIP dilution buffer, were incubated with antibodies overnight at 4°C. Antibody-protein complexes were captured with magnetic protein A/G beads, 1/10 of the IP supernatant was put aside for later analysis, beads were washed (see ChIP-seq, Supplemental Material), and antibody-protein complexes were eluted in 1× Laemmli Buffer (Laemmli 1970).

### Coimmunoprecipitation of lamin A/C and LAP2alpha

Two times 10^7^ *Tmpo* WT imMDFs were harvested in 3 mL IP buffer (20 mM Tris-HCl [pH 7.5], 150 mM NaCl, 2 mM EGTA, 2 mM MgCl_2_, 1 mM DTT, 0.5% NP-40, 1 U/mL Benzonase [Novagen], 1 mM PMSF, 1× complete protease inhibitor) and incubated on ice for 10 min. One-twentieth of the lysate was kept as “Input.” One-third of the soluble supernatant fraction was incubated with antibodies overnight at 4°C. Antibody-protein complexes were captured with magnetic protein A/G beads, 1/10 of the IP supernatant was put aside, beads were washed twice with IP buffer (w/o Benzonase) and IP samples eluted in 1× Laemmli buffer.

### Western blot analysis

Polyacrylamide gel electrophoresis was performed according to Laemmli (1970). After blotting onto nitrocellulose (Amersham), membranes were blocked with 5% BSA or Odyssey Blocking Buffer (Licor), incubated overnight with primary antibody and for 1 h with secondary antibodies.

### Immunofluorescence

One to two times 10^5^ cells on coverslips were washed once with PBS++ (1 mM CaCl_2_ and 0.5 mM MgCl_2_), fixed for 10 min with 4% formaldehyde/PBS, permeabilized with 50 mM NH_4_Cl and 0.5% Triton X-100/PBS for 10 min, and blocked with 0.5% gelatine/PBS for 15 min. Primary and secondary antibody dilutions were prepared in 0.5% gelatine/PBS and applied for 1 h at room temperature.

Samples were imaged using a Zeiss LSM 700 with a Zeiss Plan-Apochromat 63×/1.4 oil DIC objective and processed with ImageJ and Photoshop CS4.

### Antibodies

Lamin A/C (3A6-4C11, Active Motif 39287, hybridoma supernatant, 100 µL for ChIP and Co-IP, 1:500 for WB, 1:100 for IF); lamin A/C (N18, Santa Cruz sc-6215, 10 µg for ChIP and Co-IP, 1:100 for WB, 1:50 for IF); lamin B1 (Proteintech 12987-1-AP, 3 µg for ChIP, 1:1000 for WB, 1:1000 for IF); mouse LAP2alpha (MFPL Monoclonal Antibody Facility, 8C10-1H11, hybridoma supernatant, 50 µL for ChIP and Co-IP, 1:500 for WB, 1:50 for IF; [specificity validated in Supplemental Fig. 3]); LAP2alpha (245.2, Abcam ab5162, 1:1000 for IF); H3K9me3 (Abcam ab8898, 3 µg for ChIP); H3K4me3 (Millipore 07-473, 3 µg for ChIP); H3K9ac (Millipore 06-942, 3 µg for ChIP); H3K27me3 (Millipore 07-449, 3 µg for ChIP); mouse normal IgG (Millipore 12-371, 10 µg for ChIP); rabbit normal IgG (Abcam ab46540, 10 µg for Co-IP).

Secondary antibodies for WB: Licor IRDye 680RD donkey anti-mouse, 680RD donkey anti-rabbit, 800CW donkey anti-rabbit, 800CW donkey anti-goat; all 1:15000.

Secondary antibodies for IF: DyLight donkey anti-mouse 594, donkey anti-rabbit 594, goat anti-mouse 488, goat anti-rabbit 488, donkey anti-goat 549; all 1:500.

## Data access

The data from this study have been submitted to the NCBI Gene Expression Omnibus (Edgar et al. 2002) (GEO; http://www.ncbi.nlm.nih.gov/geo/) under accession number GSE70149.

## Supplementary Material

Supplemental Material
