# Supplementary material for: A-type lamins bind both hetero- and euchromatin, the latter being regulated by lamina-associated polypeptide 2 alpha
Source: Genome Res. 2016 Apr;26(4):462–73. doi: 10.1101/gr.196220.115 (PMC4817770; doi:10.1101/gr.196220.115)
Supplement: Supplemental Material [file supp_26_4_462__index.html]

A-type lamins bind both hetero- and euchromatin, the latter being regulated by lamina-associated polypeptide 2 alpha — A-type lamins bind both hetero- and euchromatin, the latter being regulated by lamina-associated polypeptide 2 alpha — Supplemental Material 

# A-type lamins bind both hetero- and euchromatin, the latter being regulated by lamina-associated polypeptide 2 alpha

## Supplemental Material

**Files in this Data Supplement:**

- Supplemental Figures and Legends.pdf
- Supplemental Material.pdf
- Supplemental Table 1.xlsx
- Supplemental Table 2.xlsx
- Supplemental Table 3.xlx
