# Supplementary material for: A-type lamins bind both hetero- and euchromatin, the latter being regulated by lamina-associated polypeptide 2 alpha
Source: Genome Res. 2016 Apr;26(4):462–73. doi: 10.1101/gr.196220.115 (PMC4817770; doi:10.1101/gr.196220.115)
Supplement: Supplemental Material [file supp_gr.196220.115_Supplemental_Figures_and_Legends.pdf]

Gesson et al. Supplemental Figure 1

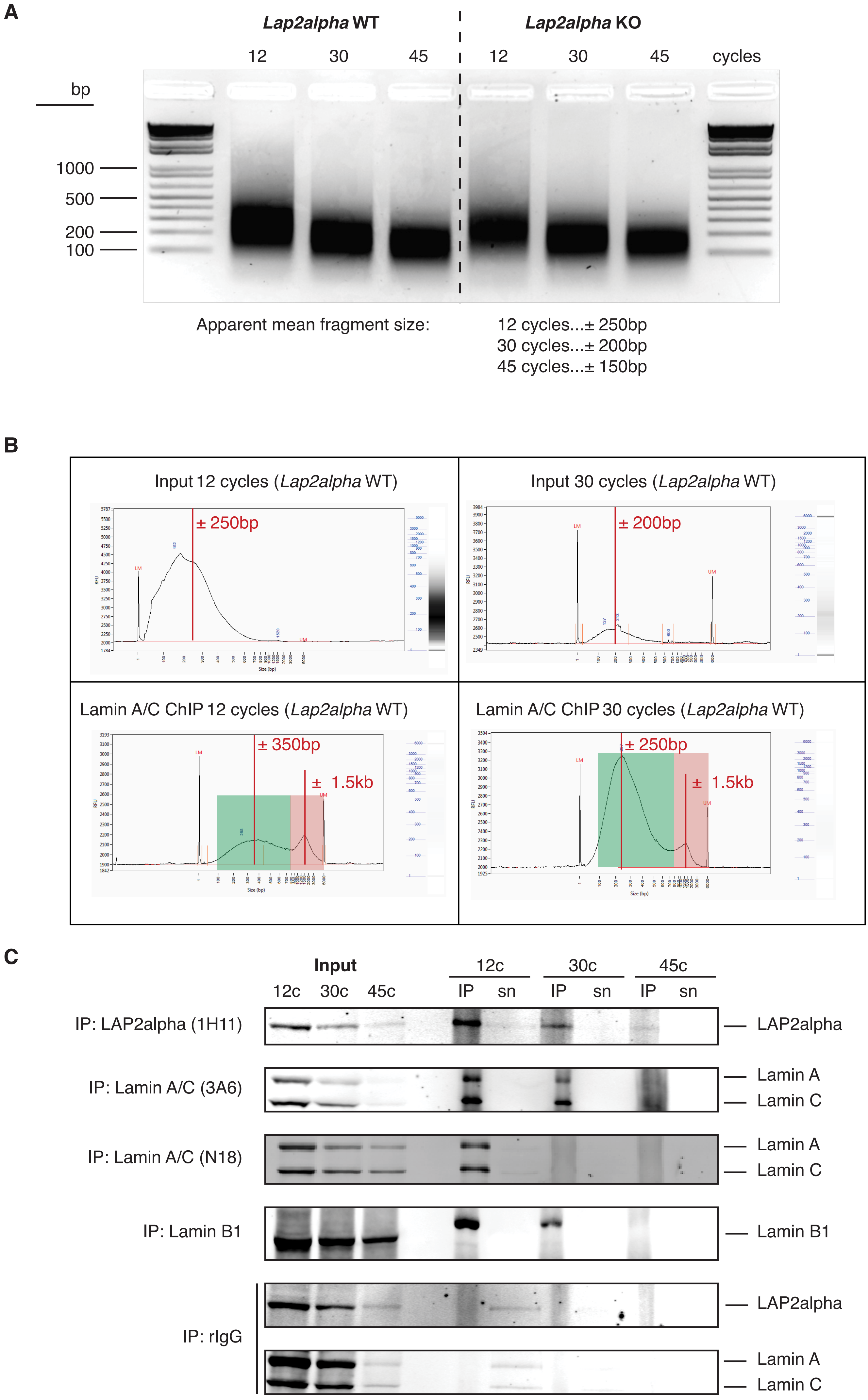

Gesson et al. Supplemental Figure 2

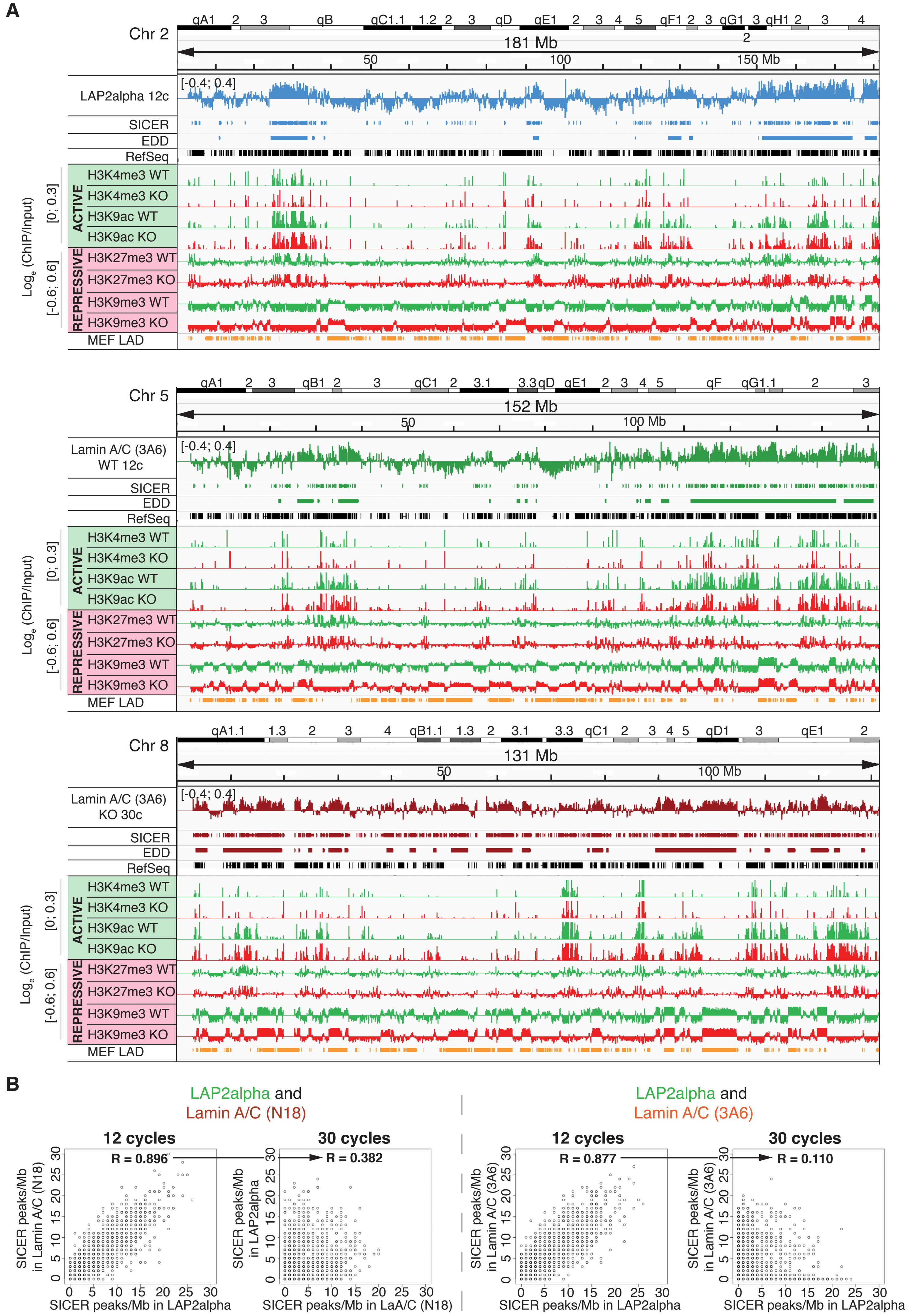

Gesson et al. Supplemental Figure 3

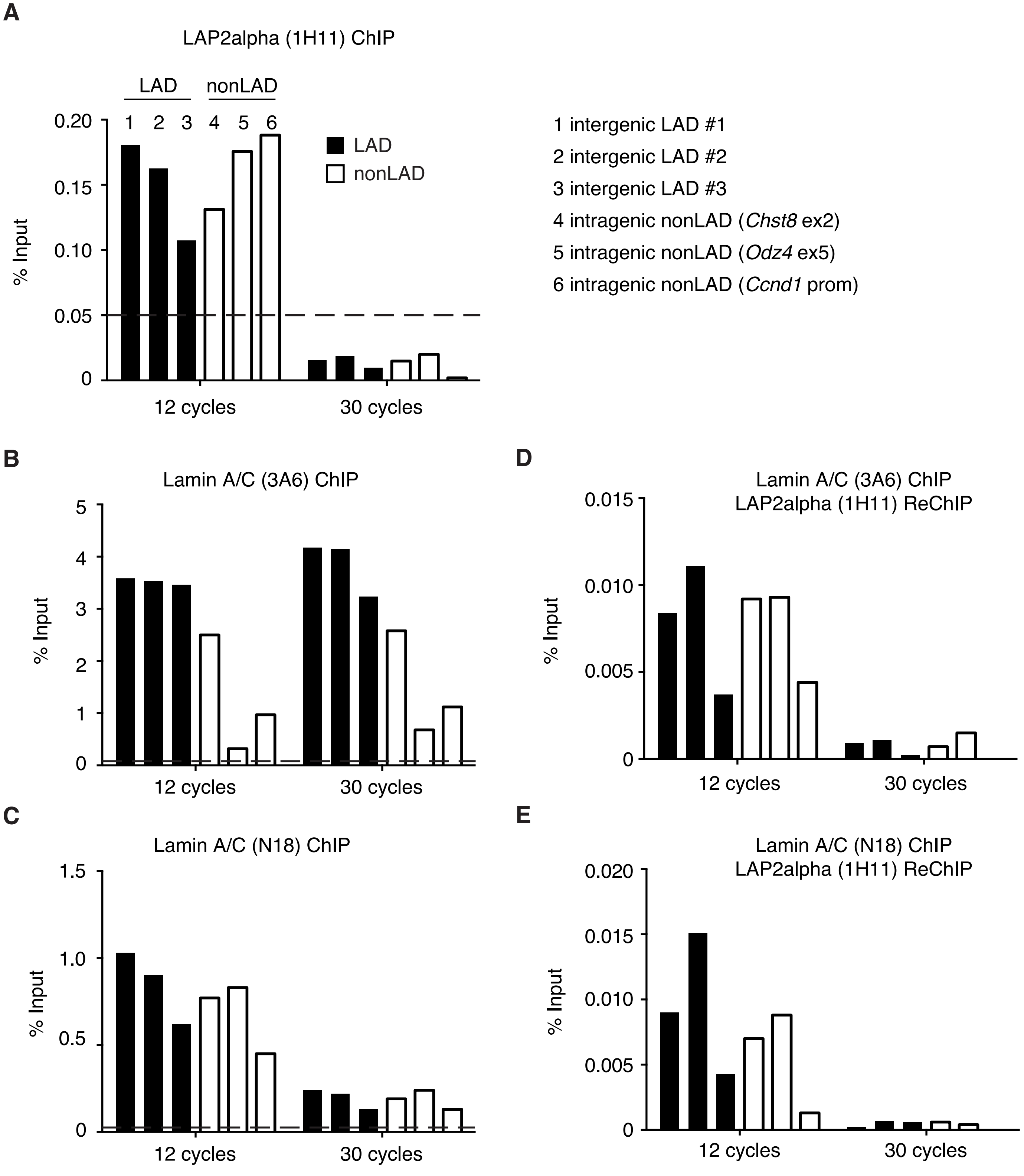

Gesson et al. Supplemental Figure 4

A

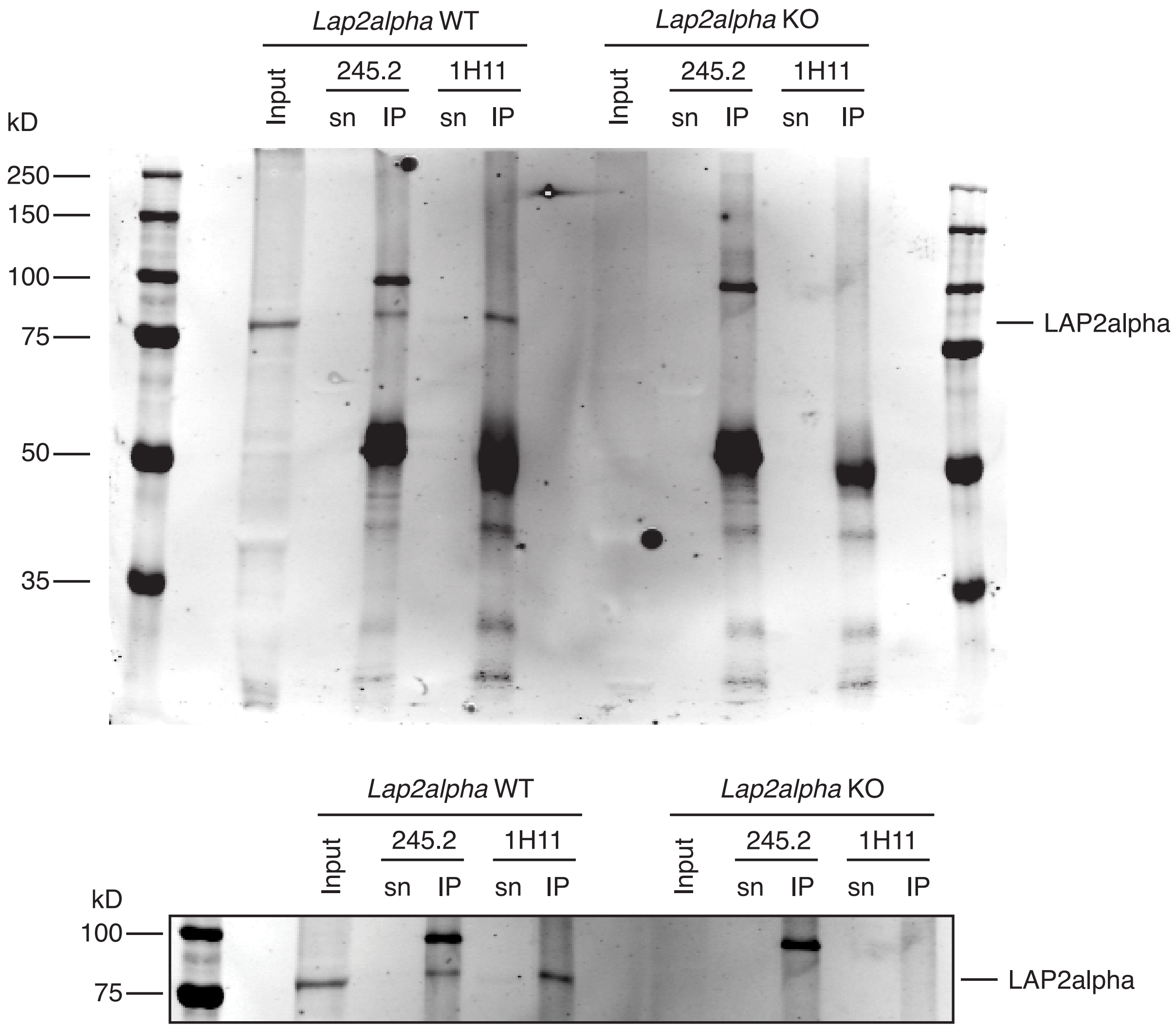

Gesson et al. Supplemental Figure 5

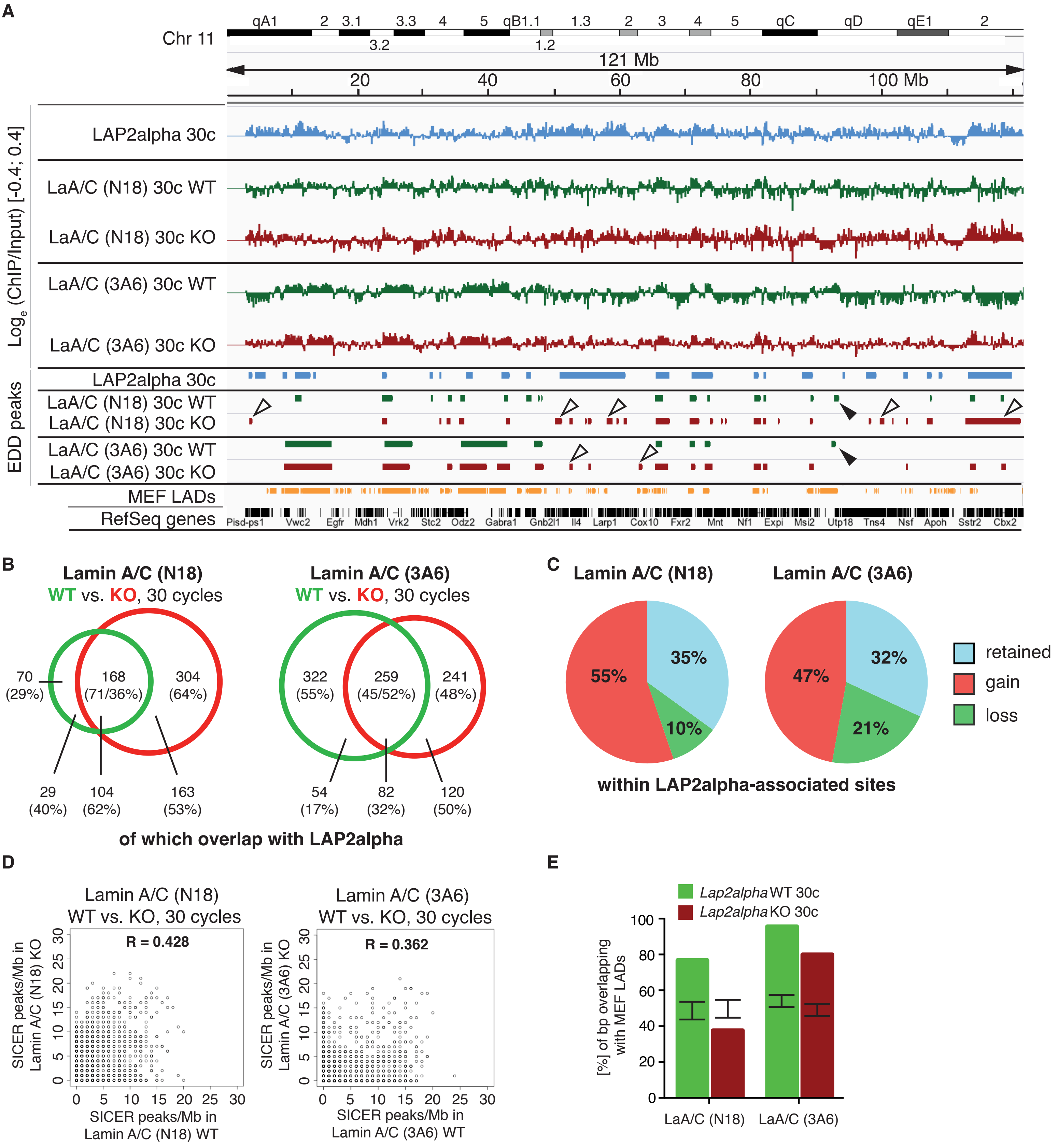

Gesson et al. Supplemental Figure 6

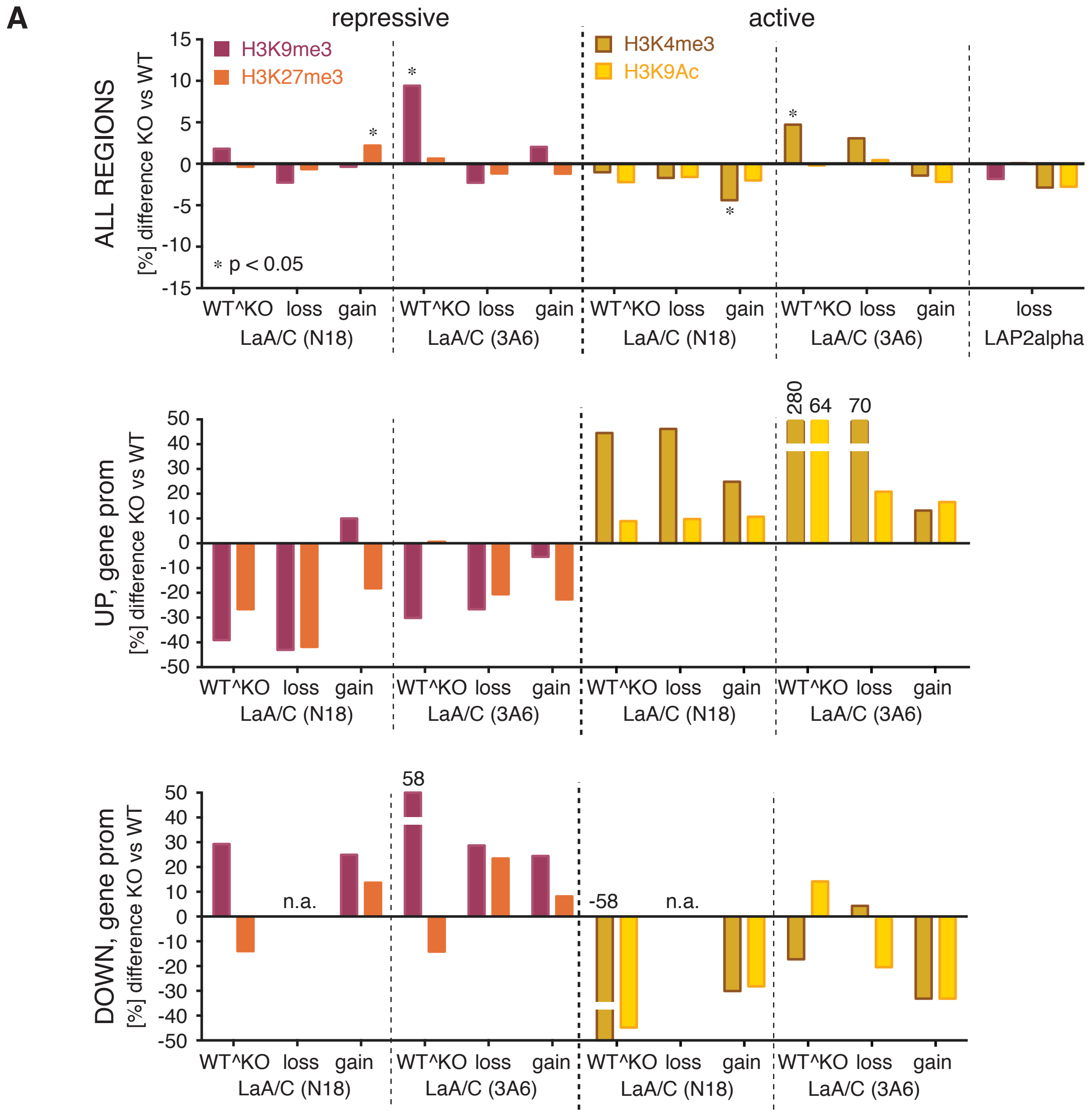

Supplemental Figure Legends

Supplemental Figure 1. Characterization of Input and ChIP material

(**A**) 2% agarose gel of Input chromatin after 12, 30 and 45 cycles of sonication. (**B**) Representative Bioanalyzer intensity profiles of input and lamin A/C (3A6) ChIP samples (*Lap2alpha* WT) after 12 and 30 cycles of sonication. Lower bp vertical red lines and numbers indicate mean fragment size. Green boxes in ChIP samples outline selected fragment size range for library preparation (100-800 bp). Higher bp vertical red lines mark the occurrence of a second peak around 1.5 kb. (**C**) Western blots of immunoprecipitations of LAP2alpha, lamin A/C, lamin B1 and rIgG from input material sonicated for 12, 30 and 45 cycles.

Supplemental Figure 2. EDD and SICER peaks correspond with each other and with active histone marks and H3K27me3 marks

(**A**) Representative IGV screenshots of $\log_e$(ChIP/Input) [-0.4;0.4], SICER peaks, EDD peaks and RefSeq genes of chromosome 2 for LAP2alpha (1H11), chromosome 5 for lamin A/C (3A6) after 12 cycles of sonication (*Lap2alpha* WT), and chromosome 8 for lamin A/C (3A6) after 30 cycles of sonication (*Lap2alpha* KO) and data of histone marks after 12 cycles of sonication (scale for active marks H3K4me3 and H3K9ac is $\log_e$ (ChIP/Input) [0;0.3] and for repressive marks H3K27me3 and H3K9me3 $\log_e$ (ChIP/Input) [-0.6;0.6]), and MEF lamina associated domains (LADs) (GSE36132) (**B**) SICER peak correlations (peaks/Mb) for indicated sample combinations as in Fig. 2D.

Supplemental Figure 3. Sequential ChIP-qPCR (ReChIP-qPCR) of Lamin A/C-3A6 or Lamin A/C-N18 and LAP2alpha

ChIPs were performed with antibodies against LAP2alpha (8C10-1H11) (**A**) and lamin A/C (3A6-4C11) (**B**) and N18 (**C**). Dashed line indicates unspecific signal in IgG control ChIP. ReChIPs were performed in a sequential manner with the first ChIP for lamin A/C (3A6-4C11 and N18) and the second ChIP for LAP2alpha (8C10-1H11) (**D**) and (**E**). Purified DNA fragments were subjected to quantitative PCR and results were calculated as % of Input. Primers and chromosomal regions are shown in Supplemental Table 4.

Supplemental Figure 4. Specific pull-down with antibodies against LAP2alpha

Immunoprecipitations for LAP2alpha were performed in *Lap2alpha* WT and KO imMDFs with a polyclonal rabbit (245.2) and a mouse monoclonal (1H11) antibody directed against LAP2alpha. Both antibodies specifically precipitated LAP2alpha, albeit the monoclonal being more efficient.

Supplemental Figure 5. Rearrangement of Lamin A/C, LAD overlap and histone mark changes upon extended sonication

(**A**) Screenshot of IGV tracks of the entire chromosome 11 (mm9) representing $\log_e$ (ChIP/Input) [-0.4;0.4], peak regions identified by EDD, MEF LADs, and the RefSeq gene track. Depicted are data of samples for LAP2alpha (1H11) and lamin A/C (precipitated with 3A6 or N18 antibodies; in *Lap2alpha* WT and KO after 30 cycles of sonication. Black arrowheads point to regions of loss of lamin A/C, white arrowheads to regions of gain of lamin A/C-association with chromatin in *Lap2alpha* KO versus *Lap2alpha* WT cells. (**B**) Venn diagrams of EDD peak overlap [Mb]

between lamin A/C in *Lap2alpha* WT and *Lap2alpha* KO after 30 cycles of sonication. Numbers below Venn diagram show overlap [in Mb and %] of lamin A/C EDD peak fractions (occurring only in WT, in WT and KO or KO only) with LAP2alpha EDD peaks. (**C**) Redistribution of lamin A/C within LAP2alpha-associated regions. Pie chart depicting percentage of lamin A/C associated regions (identified by antibodies N18 or 3A6) within the LAP2alpha-associated regions that retained, lost or gained lamin A/C binding in *Lap2alpha* KO versus WT samples. (**D**) Sicer peak correlations (peaks/Mb). (**E**) MEF LAD overlap. Degree of overlap (% of bp) of EDD peaks, obtained after 30 cycles of sonication for lamin A/C-N18 and -3A6 in *Lap2alpha* WT and KO mouse fibroblasts, with MEF LADs. Error bars indicate the interval that contains 95 % of all mean overlaps obtained through random permutation tests. All overlaps are significantly smaller or larger than expected under the null model. ($p < 10^{-4}$).

Supplemental Figure 6. Change in repressive and active histone marks upon rearrangement of lamin A/C in *Lap2alpha* KO versus WT cells after 30 cycles of sonication

(**A**) Percent difference (*Lap2alpha* KO vs. *Lap2alpha* WT) in the abundance of repressive (H3K27me3, H3K9me3) and active (H3K4me3, H3K9ac) histone marks present in all lamin A/C-N18 and -3A6 "WT^KO", "loss" and "gain" regions and in all regions of LAP2alpha loss, or present in promoter regions of up- (UP, gene prom) and downregulated (DOWN, gene prom) genes in lamin A/C-N18 and -3A6 "WT^KO", "loss" and "gain" regions after 30 sonication cycles. Asterisk (*) denotes significant change in histone mark abundance ($p < 0.05$) compared to random

permutation testing. “n.a.” denotes regions were no differentially regulated genes were found.

Supplemental Table 1. Deregulated genes in *Lap2alpha* KO versus WT

List of 616 differentially expressed genes in *Lap2alpha* KO vs. WT as determined by RNA-sequencing and analyzed with edgeR using a false discovery rate (FDR) cut-off of 0.05.

Supplemental Table 2. Deregulated genes located in regions differentially bound by lamin A/C in *Lap2alpha* KO versus WT

Subset consisting of 239 differentially expressed genes (*Lap2alpha* KO vs. WT) located in regions either gaining or losing lamin A/C interaction upon loss of LAP2alpha.

Supplemental Table 3. Sequencing read counts

Number of reads obtained by deep sequencing, after mapping, filtering and deduplication for all ChIP samples.
