# Supplementary material for: A-type lamins bind both hetero- and euchromatin, the latter being regulated by lamina-associated polypeptide 2 alpha
Source: Genome Res. 2016 Apr;26(4):462–73. doi: 10.1101/gr.196220.115 (PMC4817770; doi:10.1101/gr.196220.115)
Supplement: Supplemental Material [file supp_gr.196220.115_Supplemental_Material.pdf]

Supplemental Material

Supplemental Methods

ChIP-seq, ChIP-qPCR and sequential ChIP-qPCR (ReChIP-qPCR)

For ChIP, asynchronously growing *Lap2alpha* WT and *Lap2alpha* KO imMDFs were washed twice with ice-cold phosphate-buffered saline (PBS++, supplemented with 1 mM Ca2+, 0.5 mM Mg2+) and cross-linked in 1% formaldehyde / PBS++ for 10 min at room temperature. Cross-linking was stopped by the addition of glycine to a final concentration of 125 mM. Cells were washed twice with ice-cold PBS++ and scraped into ice-cold PBS++ containing 1 mM phenylmethylsulfonyl fluoride (PMSF) and 1x complete protease inhibitor, EDTA-free (Roche, Ref.no: 05056489001). After centrifugation at 2000 x g for 5 min at 4 °C, the cell pellet was resuspended in wash buffer I (10 mM HEPES [pH 7.5], 0.25% Triton X-100, 10 mM EDTA, 0.5 mM EGTA, 1 mM PMSF, 1x complete protease inhibitor), incubated on ice for 10 min, and centrifuged. Washing and incubation was repeated with wash buffer II (10 mM HEPES [pH 7.5]), 0.2 M NaCl, 1 mM EDTA, 0.5 mM EGTA1 mM PMSF, 1x complete protease inhibitor). Following centrifugation, nuclei-containing pellets were resuspended in lysis buffer (50 mM Tris-HCl [pH 8.1], 10 mM EDTA, 1% sodium dodecyl sulfate [SDS] 1 mM PMSF, 1x complete protease inhibitor) and sonicated with a Bioruptor® Plus (Diagenode) for 12 or 30 cycles (30 sec ON/OFF, on “HIGH” setting, 4 °C) with water exchange every 3-5 cycles. For histone mark ChIPs 12 cycles of sonication were applied. The sheared chromatin was cleared twice by centrifugation at 14.000 x g for 10 min at 8 °C. Chromatin in the supernatant is referred to as input. 50 µg of chromatin (derived from approximately $5x10^6$ cells) were used for ChIP. Input chromatin was diluted 1:10 in ChIP dilution buffer (16.7 mM Tris-HCl [pH 8.1], 167 mM NaCl, 1.2 mM EDTA, 1.1 % Triton X-100, 0.01%

SDS), antibodies were added and incubated over night on a rotary shaker at 4 °C. For harvesting chromatin- antibody complexes, 30 µl of magnetic protein A/G beads (Pierce, #88803) were washed twice in ChIP dilution buffer, resuspended in the original volume and added to the ChIP samples for 4-5 hours (rotary shaker, 4 °C).

The magnetic beads were collected with a magnetic rack and washed once each with RIPA buffer (50 mM Tris-HCl [pH 8.0], 150 mM NaCl, 0.1% SDS, 0.5% sodium deoxycholate, 1% NP-40), High Salt buffer (50 mM Tris [pH 8.0], 500 mM NaCl, 0.1% SDS, 1% NP-40), LiCl buffer (50 mM Tris [pH 8.0], 25 mM LiCl, 0.5% sodium deoxycholate, 1% NP-40), and two times with TE buffer (10 mM Tris [pH 8.0], 1 mM EDTA). Chromatin antibody complexes were eluted from the magnetic protein A/G beads by addition of 200 µl 2% SDS, 0.1 M $NaHCO_3$, and 10 mM dithiothreitol to the pellet. Cross-links were reversed by addition of 0.05 volume of 4 M NaCl and incubation of the eluted samples for 6 h to over night at 65°C. After addition of 0.025 volume of 0.5 M EDTA and 0.05 volume of 1 M Tris-HCl (pH 6.5), proteinase K (4 µg; Ambion, Cat.No. AM2546) digestion was performed for 1 h at 55°C.

Eluted DNA was purified with the 'ChIP DNA Clean & ConcentratorTM' kit (Zymoresearch, Cat.No. D5205) according to the manufacturers' protocol.

For ChIP-seq the sequencing libraries were prepared from DNA fragments ranging from 100 – 800 bp by the Vienna Biocenter CSF NGS unit (www.csf.ac.at) using the NEBNext Library Prep Reagent Set for Illumina according to the manufacturer's instructions (NEB; Cat.No. E6000S) and sequenced either on an Illumina HiSeq 2000 or HiSeq 2500 at the CSF NGS unit.

For ChIP-qPCR cells were harvested, obtained chromatin was processed and ChIPs were performed as described. ReChIP-qPCR was performed according to (Truax and

Greer 2012) as follows: 100 µg of chromatin were used for the first ChIP. After the first ChIP with lamin A/C antibodies the magnetic protein A/G beads were washed three times with ReChIP wash buffer (50 mM Tris [pH 8.0], 500 mM NaCl, 0.1% SDS, 1% NP-40, 2 mM EDTA), chromatin-antibody complexes were eluted in ReChIP elution buffer (10 mM Tris [pH 8.0], 2% SDS, 15 mM DTT) at 37°C for 30 min and subsequently diluted 1/20 in ChIP dilution buffer (16.7 mM Tris-HCl [pH 8.1], 167 mM NaCl, 1.2 mM EDTA, 1.1 % Triton X-100, 0.01% SDS). LAP2alpha antibody was added and incubated over night on a rotary shaker at 4 °C. Second round chromatin-antibody complexes were captured, washed and eluted and DNA was purified as for ChIP-seq. Purified DNA was used for quantitative PCR using KAPA SYBR FAST Universal 2x qPCR master mix (Peqlab, 07-KK4600-01) and results were calculated as % of Input.

ChIP-seq data analysis

To quantify the degree of overlap between EDD peaks of two ChIP samples (A and B) we counted the number of base pairs covered by peaks in only the first (A only), only the second (B only) or both samples (A and B) using BEDTools (Quinlan and Hall 2010). The resulting numbers were visualized in Venn diagrams. To compare the lamin A/C, LAP2alpha and lamin B1 regions to published LAD annotations we computed for each set of EDD peaks the percentage of base pairs that overlap with ciLADs and MEF LADs obtained from the GEO database (see Data viewing). In contrast to lamin and LAP2alpha data presented here, ciLADs and MEF LADs are based on peaks called with a two-state HMM from DamID tiling arrays. However, the large overlap between MEF LADs and our lamin B1 data shows, that overall both protocols produce comparable results. Similar to (Gel et al. 2015), the statistical

significance of an overlap was assessed by comparing to a null distribution of overlaps obtained from 10,000 random permutations. For all random permutation tests, we excluded all “gap” regions on the genome (retrieved from UCSC Genome Browser). Genes per megabase were computed individually for each peak. Only genes that overlap by at least 50% with a peak were considered. The final number of overlapping genes for each peak was then normalized to its length. P-values were computed using the Wilcoxon Rank Sum Test.

Peaks detected by SICER are much shorter than typical lamin and LAP2alpha binding domains (Lund et al. 2014). Thus, regions of enrichment are covered by a high number of short peaks instead of one long peak. Since the exact position of these short peaks within an enriched region can vary between samples, simple overlaps are not suitable to quantify similarity in SICER peak coverage between lamin and LAP2alpha samples. Therefore, we divided the genome into non-overlapping 1 Mb bins and used the number of SICER peaks per bin to measure the similarity between samples. To this end, each bin *b* is represented by two peak counts (x_b, y_b), where x_b and y_b represent the number of SICER peaks in sample A and B, respectively. For all bins the peak counts were illustrated in a scatter-plot. Finally, we computed the Pearson Correlation coefficient between the peak counts per bin in sample A and sample B, and used this as a measure of similarity of SICER peak coverage.

Histone mark abundance

To determine histone mark abundance on promoters, we defined promoters as regions in the genome that extend from 2,200 bp upstream to 500 bp downstream of transcription start sites (TSS), similar to Lund et al. (Lund et al. 2013). For each gene we counted the number of histone ChIP and the number of input reads that mapped to

its promoter region. After normalizing the read counts by the total library size, we computed the log ratio of the normalized ChIP count and the normalized input count. The same approach was used to measure histone mark abundance in lamin A/C and LAP2alpha binding regions. Instead of individual promoter regions, we used all regions covered by EDD peaks in a sample to compute log ratios. In addition, we computed the log ratio separately for regions that gained, lost or maintained lamin A/C or LAP2alpha binding in *Lap2alpha* KO cells. For data in Figure 1E in main manuscript, the significance of differences in histone mark enrichment between 12 and 30 cycle samples was assessed using the Wilcoxon Rank Sum Test. For data in Figure 4B and Supplemental Figure 6 in main manuscript, the significance of differences in histone mark enrichment changes was assessed by random permutation testing (n = 10,000).

RNA sequencing and analysis

10 μg of purified total RNA (>200 bp) were depleted of rRNA and fragmented by hydrolysis (40 mM TrisOAc at pH 8.2, 100 mM KOAc, 150 mM MgOAc) at 94°C for 3 min. First-strand cDNA synthesis was carried out using the SuperScript III Reverse Transcriptase kit (Invitrogen) with random hexamers priming (Applied Biosystems) in the presence of actinomycin D (5 ng/μl). Second-strand cDNA was synthesized using DNA Pol I, DNA ligase (Invitrogen), and RNase H (NEB) with random hexamers (Applied Biosystems) in the presence of dUTP. The sequencing libraries were prepared by the Vienna Biocenter CSF NGS unit using the NEBNext Library Prep Reagent Set for Illumina (NEB), multiplexed (2 samples/lane), and sequenced on HiSeq 2000 (Illumina) at the CSF NGS unit. RNA-seq data analysis was done as described by Anders et al. (Anders et al. 2013). Briefly, after adapter

removal using cutadapt (Martin 2011), reads were mapped to the mouse genome (NCBI37/mm9 annotation from July 2007) using TopHat2 (Kim et al. 2013) version 2.0.9 and RefSeq gene annotations retrieved from the UCSC Genome Browser. Htseq-count (Anders et al. 2015) was used to create a count table taking into account only reads with a mapping quality larger than 20. All annotated transcripts that show at least one mapped read per one million sequenced reads (counts per million) in both replicates were tested for differential expression using edgeR (Robinson et al. 2010) and a false discovery rate (FDR) cutoff of 0.05. All significantly up- or down-regulated genes are listed in Supplemental Table 1. Significant changes in expression in Lamin A/C gain, loss and WT^KO regions were detected by comparing the actual log2 fold change to fold changes obtained from 10,000 random permutations of differentially expressed genes. ($p < 0.05$).

References

Anders S, McCarthy DJ, Chen Y, Okoniewski M, Smyth GK, Huber W, Robinson MD. 2013. Count-based differential expression analysis of RNA sequencing data using R and Bioconductor. *Nat Protoc* **8**: 1765-1786.

Anders S, Pyl PT, Huber W. 2015. HTSeq--a Python framework to work with high-throughput sequencing data. *Bioinformatics* **31**: 166-169.

Gel B, Diez-Villanueva A, Serra E, Buschbeck M, Peinado MA, Malinverni R. 2015. regioneR: an R/Bioconductor package for the association analysis of genomic regions based on permutation tests. *Bioinformatics* doi:10.1093/bioinformatics/btv562.

Kim D, Pertea G, Trapnell C, Pimentel H, Kelley R, Salzberg SL. 2013. TopHat2: accurate alignment of transcriptomes in the presence of insertions, deletions and gene fusions. *Genome Biol* **14**: R36.

Lund E, Oldenburg AR, Collas P. 2014. Enriched domain detector: a program for detection of wide genomic enrichment domains robust against local variations. *Nucleic Acids Res* **42**: e92.

Lund E, Oldenburg AR, Delbarre E, Freberg CT, Duband-Goulet I, Eskeland R, Buendia B, Collas P. 2013. Lamin A/C-promoter interactions specify chromatin state-dependent transcription outcomes. *Genome Res* **23**: 1580-1589.

Martin M. 2011. Cutadapt removes adapter sequences from high-throughput sequencing reads. *EMBnetjournal* **17**.

Quinlan AR, Hall IM. 2010. BEDTools: a flexible suite of utilities for comparing genomic features. *Bioinformatics* **26**: 841-842.

Robinson MD, McCarthy DJ, Smyth GK. 2010. edgeR: a Bioconductor package for differential expression analysis of digital gene expression data. *Bioinformatics* **26**: 139-140.

Truax AD, Greer SF. 2012. ChIP and Re-ChIP assays: investigating interactions between regulatory proteins, histone modifications, and the DNA sequences to which they bind. *Methods Mol Biol* **809**: 175-188.

Supplemental Table 4. Primers used for (Re)ChIP-qPCR

#	Type of region	Genomic location		Primer sequence (5' to 3')	Annealing Tempera-ture
1	Intergenic LAD	chr4:121406388-121406519	forw	CACCTCCATTGGATTTTGCT	64 ºC
			rev	GCAATGCAAAAGCTGACAAA	
2	Intergenic LAD	chr4:121766869-121766966	forw	CTGAGGCCATCTCTGCTAGG	64 ºC
			rev	AGAAGGCAGCCCTTTGTCTT	
3	Intergenic LAD	chr7:55155343-55155441	forw	AAGGAGGGACCCTGAGAGAA	64 ºC
			rev	GGAAGACAGATGCTGGAAGC	
4	intragenic nonLAD (*Chst8* gene, exon2)	chr7:35531566-35531649	forw	TGCGTTTCTTCCTGTCCTCT	62 ºC
			rev	GCAAACTGGAGCTCTGAACC	
5	intragenic nonLAD (*Odz4* gene, exon5)	chr7:103774934-103775013	forw	GTCATCTGCTGCACTTTGGA	64 ºC
			rev	AGTTTTCCACACAGCCATCC	
6	intragenic nonLAD	chr7:152125670-152125820	forw	CCGGCTTTGATCTCTGCTTA	62 ºC
			rev	CGCTGTACTGCCGGTCTC	
